# Comparative phylogenomic analyses of teleost fish Hox gene clusters: lessons from the cichlid fish *Astatotilapia burtoni*

**DOI:** 10.1186/1471-2164-8-317

**Published:** 2007-09-10

**Authors:** Simone Hoegg, Jeffrey L Boore, Jennifer V Kuehl, Axel Meyer

**Affiliations:** 1 Lehrstuhl für Evolutionsbiologie und Zoologie, Department of Biology, University of Konstanz, 78457 Konstanz, Germany; 2 Program in Evolutionary Genomics, DOE Joint Genome Institute and Lawrence Berkeley National Laboratory, and University of California, Berkeley, California 94720, USA; 3 SymBio Corporation, 1455 Adams Drive, Menlo Park, CA 94025, and University of California, Berkeley, California 94720, USA

## Abstract

**Background:**

Teleost fish have seven paralogous clusters of Hox genes stemming from two complete genome duplications early in vertebrate evolution, and an additional genome duplication during the evolution of ray-finned fish, followed by the secondary loss of one cluster. Gene duplications on the one hand, and the evolution of regulatory sequences on the other, are thought to be among the most important mechanisms for the evolution of new gene functions. Cichlid fish, the largest family of vertebrates with about 2500 species, are famous examples of speciation and morphological diversity. Since this diversity could be based on regulatory changes, we chose to study the coding as well as putative regulatory regions of their Hox clusters within a comparative genomic framework.

**Results:**

We sequenced and characterized all seven Hox clusters of *Astatotilapia burtoni*, a haplochromine cichlid fish. Comparative analyses with data from other teleost fish such as zebrafish, two species of pufferfish, stickleback and medaka were performed. We traced losses of genes and microRNAs of Hox clusters, the medaka lineage seems to have lost more microRNAs than the other fish lineages. We found that each teleost genome studied so far has a unique set of Hox genes. The *hoxb7a *gene was lost independently several times during teleost evolution, the most recent event being within the radiation of East African cichlid fish. The conserved non-coding sequences (CNS) encompass a surprisingly large part of the clusters, especially in the HoxAa, HoxCa, and HoxDa clusters. Across all clusters, we observe a trend towards an increased content of CNS towards the anterior end.

**Conclusion:**

The gene content of Hox clusters in teleost fishes is more variable than expected, with each species studied so far having a different set. Although the highest loss rate of Hox genes occurred immediately after whole genome duplications, our analyses showed that gene loss continued and is still ongoing in all teleost lineages. Along with the gene content, the CNS content also varies across clusters. The excess of CNS at the anterior end of clusters could imply a stronger conservation of anterior expression patters than those towards more posterior areas of the embryo.

## Background

Genome duplications [1,2] and regulatory evolution [3-5] are thought to be two major genomic evolutionary mechanisms that are, at least partly, responsible for the increased diversity of vertebrates compared to their chordate relatives. Genome and gene duplications can provide the raw material on which evolution can act since they lead to redundant gene copies that are freed up to evolve novel gene functions [1,6].

Sequence data from complete genomes of tetrapods such as mouse, frog and human as well as from invertebrates such as *Caenorhabditis elegans *and *Drosophila melanogaster *show that many gene families tend to be larger in vertebrates [7-9]. Synteny data demonstrated that the most likely scenario for the increased size of gene families are two consecutive rounds of genome duplication, the so-called 2R-hypothesis [10-12]. Genomic data from zebrafish and pufferfish showed that many genes were duplicated before the divergence of those two species representing the major fish orders Neoteleostei and Ostariophysii [13-15]. More recently, syntenic data from zebrafish, medaka and pufferfish further confirmed the existence of an additional genome duplication event within the ray-finned fish lineage, the fish-specific genome duplication (FSGD) [16-18]. The most recent comparative genomic analyses also support the FSGD and found that the majority of genes was duplicated around 320–350 mya [19,20]. Moreover, studies on individual nuclear genes and gene families propose a timing of the duplication preceding the diversification of teleosts [15,21-23].

One of the earliest and best-studied examples for duplicated chromosomal regions is the clusters of Hox genes [21,24,25]. Hox genes are transcription factors, characterized by their DNA binding domain, the homeodomain. They were first discovered in *Drosophila *as the target of homeotic mutation, meaning the change of the segmental identity, as in the bithorax phenotype [26]. One special feature of Hox genes is their arrangement in genomic clusters. While invertebrates have a single cluster that can be interrupted as in *Drosophila *species [27] or dispersed through the genome as in urochrodates [28,29] and nematodes [30], tetrapods such as human or frogs all have four clusters [reviewed in [31,32]], as do cartilaginous fish [33]. Even invertebrates closely related to vertebrates, such as the cephalochordate *Branchiostoma *[25,34] have a single cluster, which in the case of the sea urchin, is also rearranged [35]. Due to the fish-specific genome duplication, extant fish have seven Hox clusters, with alternate cluster loss in Ostariophysi (HoxDb in zebrafish) [36] and Acanthopterygii (HoxCb in pufferfish, medaka, cichlid) [18,37-40]. The additional clusters, however, are not exactly equivalent with the homologous genes of tetrapods, but have experienced independent losses of genes [31], making the teleost clusters much more variable in gene content than those of tetrapods. So far, all of the fish that have been studied showed differences in gene content among their Hox clusters [18,31,37,41].

Individual gene loss after gene or genome duplication events is common and can occur even long after the duplication [42-44]. Interestingly, some functional categories such as signal transducers and transcriptional regulators tend to retain more members than most gene families created by duplication [45-47]. The reasons and mechanisms for these differences in rate of gene loss among different functional groups remain incompletely understood, but current theories propose a link of equimolar amounts of different regulatory genes (gene balance hypothesis) [48].

The other main genomic source for evolutionary change is thought to be the evolution of regulatory sequences, so called regulatory evolution [49]. Also for this major type of evolutionary change, Hox genes are an often-studied example [50-54]. The clustered nature of Hox genes facilitates comparison of orthologous and paralogous sequences and the high degree of conservation allows for identification and detailed analyses of evolutionary events in regulatory sequences. Vertebrate Hox clusters are almost free of repetitive elements [55] which adds further tractability to the study of regulatory evolution. Hox genes play an important role in the specification of the primary body axis [56] as well as in later ontogenetic processes demanding highly specific regulation such as limb development [57].

Conserved Non-coding Sequences (CNS) in Hox clusters have been intensely studied previously, both in terms of content and cluster identity [53,58,59] as well as their evolutionary rates in duplicated clusters [50,54]. The intergenic regions of Hox clusters are enriched for CNS and it has been argued that this abundance of cis- and trans-regulatory elements is the main reason for cluster conservation since neighboring genes share regulatory elements. However, it is unclear how strong this "gluing effect" of regulatory elements is for the cohesion of Hox genes in clusters since Hox clusters in at least some invertebrates can be split without apparent loss of function [27]. One possible source of the higher plasticity of the invertebrate cluster is the presence of repetitive DNA while in vertebrates, there is strong selection acting against it [55].

Actinopterygian (ray-finned) fishes not only encompass more than half of all vertebrate species (about 27,000) [60], but also display a huge variety of body shapes. One particular species-rich, monophyletic group of derived teleosts is the Euteleostei, currently ranked as one of the four subdivisions of the Teleostei, along with the more basal groups, Osteoglossomorpha, Elopomorpha, and Clupeomorpha [61-65]. The Euteleostei comprise approximately 25,000 species, of which 17,000 are Neoteleosts (e.g., pufferfishes, medaka, cichlids, and stickleback) and 8,000 Ostariophysi (e.g. zebrafish)[60]. Among the Neoteleostei, most species are classified as Perciformes (about 10,000 species), this however is a polyphyletic assembly, with at least five lineages [62]. One family of the Perciformes are cichlid fishes (Family Cichlidae), with more than 2,500 species; almost ten percent of all fish species are cichlids. Of particular interest is the immense species richness of the adaptive radiations of the East African Lakes Victoria, Malawi and Tanganyika which are made up each of several hundred endemic species each [66,67]. The species flocks of Lake Victoria as well as of Lake Malawi are monophyletic and hundreds of species arose within less than 100,000 years in the case of the Lake Victoria species flock – the fastest known rates of speciation [66,68].

One of the most intriguing questions now is, whether there is a genetic basis for this astonishing speciation rate and the enormous morphological diversity cichlids show. Since these events occur very rapidly, changes involving regulatory pathways are likely to be involved. Hox clusters provide a good starting point for a genomic investigation of this kind due to their clustered structure, which can be easily homologized with other species, and because of their known high content of CNS [59], combined with their key role in early and later development.

During the last few years, complete genomic sequences have become available for many species, including several teleost fishes. Some species were selected either for their small genome size (e.g., the pufferfishes *Takifugu rubripes *and *Tetraodon nigroviridis*) [18,69] or because they are model organisms of developmental research (*Danio rerio, Oryzias latipes*) or speciation (*Gasterosteus aculeatus*)[70]. The construction of large insert libraries such as those in BAC or fosmid vectors, make it possible to study genomic regions also for species for which a genome project is (not yet) available [23,51,71].

In this study, we sequenced the Hox-cluster containing BAC clones from the East African cichlid *Astatotilapia burtoni *[72]. We performed a phylogenetic analysis with concatenated coding sequences and investigated gene and microRNA loss in the clusters as well as content of conserved non-coding sequences in the *A. burtoni *clusters in comparison with all available teleost Hox gene clusters. The clusters show a tendency to preserve a higher amount of CNS towards the 'anterior' end of the cluster, the region which is fundamentally involved in development of the head [73,74], while the 'posterior' part contains more variation in regulatory elements.

## Results

**Figure 1:**
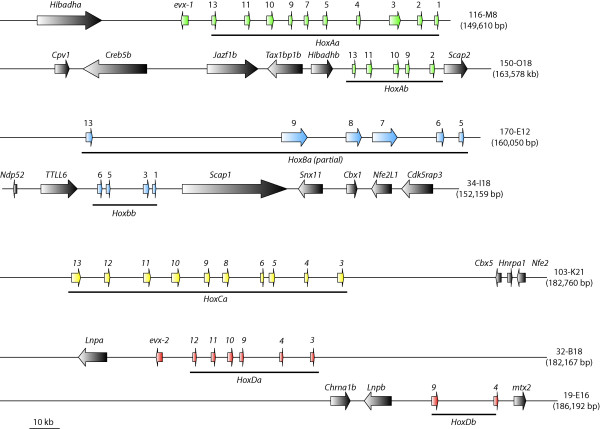
**Sequenced BAC clones and the annotated genes drawn to scale**. Hox and Evx genes are shown in color, neighboring genes are drawn in black. Abbreviations used are according to [38], the surrounding genes are identical to those found in *Takifugu rubipes*. The HoxBa cluster is incomplete, sequence data stops at 12 kb downstream of *hoxb5a*. Sequence data for the remaining four Hox genes and the non-coding regions of remaining parts of the cluster have been gathered by PCR, indicating that the clustered structure still exists.

We screened the BAC library of *Astatotilapia burtoni *[72] for clones containing Hox clusters using specific-probes. Fragments spanning the intron were used as probes to avoid non-specific cross-reactions. Positive clones obtained by these screens were checked with specific primers for the 5' and 3' most genes of a cluster (e.g. *evx-2 *and *hoxd3a*) to confirm that they contain complete clusters. Clones containing all Hox genes of a cluster were shotgun sequenced. In this way, we obtained seven BAC clones which contain complete *HoxAa*, *HoxAb*, *HoxBb*, *HoxCa*, *HoxDa *and *HoxDb *clusters as well as the 5' part of the *HoxBa *cluster spanning the region from *hoxb13a *to *hoxb5a *(Figure 1).

The clone 116-M8 (149.6 kb) contained the complete HoxAa cluster from *hoxa13a *to *hoxa1a *plus the related *evx1 *gene. 5' to *evx1 *we also identified the complete coding sequence of *Hibadha *(3-hydroxyisobutyrate dehydrogenase a). This is in agreement with the gene order in the *Takifugu rubripes *genome. The complete Hox containing region of the HoxAa cluster (including *evx1*) spans 86 kb.

For the *HoxAb *cluster, we selected clone 150-O18 (164.6 kb) for sequencing. The genes in this genomic region are tightly packed; this is true for the Hox genes as well as the surrounding genes. Also here the adjacent genes are orthologous to pufferfish sequences (*cpv1 *(carboxypeptidase vitellogenic-like), *creb5b *(cAMP responsive element binding protein 5), *jazf1b *(juxtaposed with another zinc finger protein 1), *tax1bp1b *(Tax1 binding protein 1), *hibadhb *(3-hydroxyisobutyrate dehydrogenase b), and *skap2 *(Src family associated phosphoprotein 2)). The *HoxAb *cluster is a small cluster both in terms of number of genes as well as intergenic regions and has a size of only 27 kb.

The *HoxBa *cluster is the largest in the genome of *A. burtoni *and we sequenced clone 170-E12 (160.1 kb), which contains the 5' part of the cluster from *hoxb13a *to *hoxb5a*. Despite intense screening of the BAC library with probes for the 3'genes (*hoxb4a*, *hoxb3a*, *hoxb2a*, *hoxb1a*), we could not identify a BAC clone containing this region in our library. We were able to amplify those genes from genomic DNA however. Therefore, they are not lost from the genome of *Astatotilapia burtoni*, but were apparently not contained in our BAC library. Similar to *Gasterosteus aculeatus*, *Astatotilapia burtoni *also has a large intergenic region (63 kb) containing repetitive elements between *hoxb13a *and *hoxb9a*. While this region looks "normal" in both pufferfish species as well as in medaka and zebrafish, this region in tetrapods also appears to be "decaying". So far, no *hoxb13 *gene from frog could be identified [31,75] and in the human cluster, two non-Hox genes are situated between *HoxB13 *and *HoxB9 *(PRAC, LOC729146). The increase in size of the *HoxBa *cluster relative to that of the other Hox clusters also affects the size of its intergenic and the intronic regions. Although the *hoxb7a *gene of *Astatotilapia burtoni *was identified and annotated without problems, the coding sequence contains a stop codon at the beginning of the coding sequence, rendering it a pseudogene. However, in the other cichlid species studied so far, *Oreochromis niloticus*, *hoxb7a *has a completely intact coding sequence indicating that the non-functionalization of this gene in *A. burtoni *occurred within only the approximately last seven million years since the two species last shared a common ancestor.

The paralogous cluster of the "giant" HoxBa, is the "dwarf" HoxBb cluster, which was identified within BAC clone 34-B18 (152.2 kb). It contains only four Hox genes (*hoxb6b*, *hoxb5b*, *hoxb3b*, and *hoxb1b*) and spans only about 20 kb. Apart from the Hox genes themselves, the genes surrounding the HoxBb cluster are also densely packed. The clone contains also a partial sequence of *ndp52 *(nuclear domain 10 protein 52) and the complete sequences of *TTLL6 *(tubulin tyrosine ligase-like family member 6), *scap1 *(Src family associated phosphoprotein 1), *snx11 *(sorting nexin 11), *cbx1 *(chromobox-like 1), *nfe2l1 *(nuclear factor erythroid derived 2-like 1), and *cdk5rap3 *(CDK5 regulatory subunit associated protein 3) (Figure 1).

Clone 103-K21 (182.8 kb) contains the complete HoxCa cluster and three additional genes 3' of the cluster (*cbx5 *(chromobox-like 5), *hnrpa1 *(heterogeneous nuclear ribonucleoprotein A1), and *nfe2 *(nuclear factor erythroid-derived *2*)). Also here the order of the neighbouring genes is the same as in *Takifugu rubripes*. This also confirms that there are no further Hox genes downstream of *hoxc3a *in cichlids, while in zebrafish *hoxc1a *was retained. The complete length of the HoxCa cluster in *Astatotilapia burtoni *from *hoxc13a *to *hoxc3a *is 91 kb.

The HoxDa cluster was sequenced from clone 32-B18 (182.2 kb). This Hox cluster only spans 53 kb from *evx2 *to *hoxd3a*, and the surrounding sequences contain only one more gene, *lnpa *(lunapark a).

From clone 19-E16 (186.2 kb), the sister cluster HoxDb was sequenced. Two upstream genes (*chrna1b *(cholinergic receptor nicotinic alpha polypeptide) and *lnpb *(lunapark b)) confirm that there are only two Hox genes in this cluster, *hoxd9b *and *hoxd4b*, and that *hoxd11b*, which is present in HoxDb clusters of stickleback and the two pufferfishes was lost in cichlids. Downstream of *hoxd4b*, we identified the complete coding region of *mtx2 *(metaxin 2).

In general, the neighboring genes that were identified were orthologous to those in *Takifugu rubripes*. From other fish genomes, the neighbouring genes were not identified in such detail. Based on blast hits adjacent to the Hox genes, it appears that gene order is conserved generally in teleost species.

### Phylogenetic analyses

**Figure 2:**
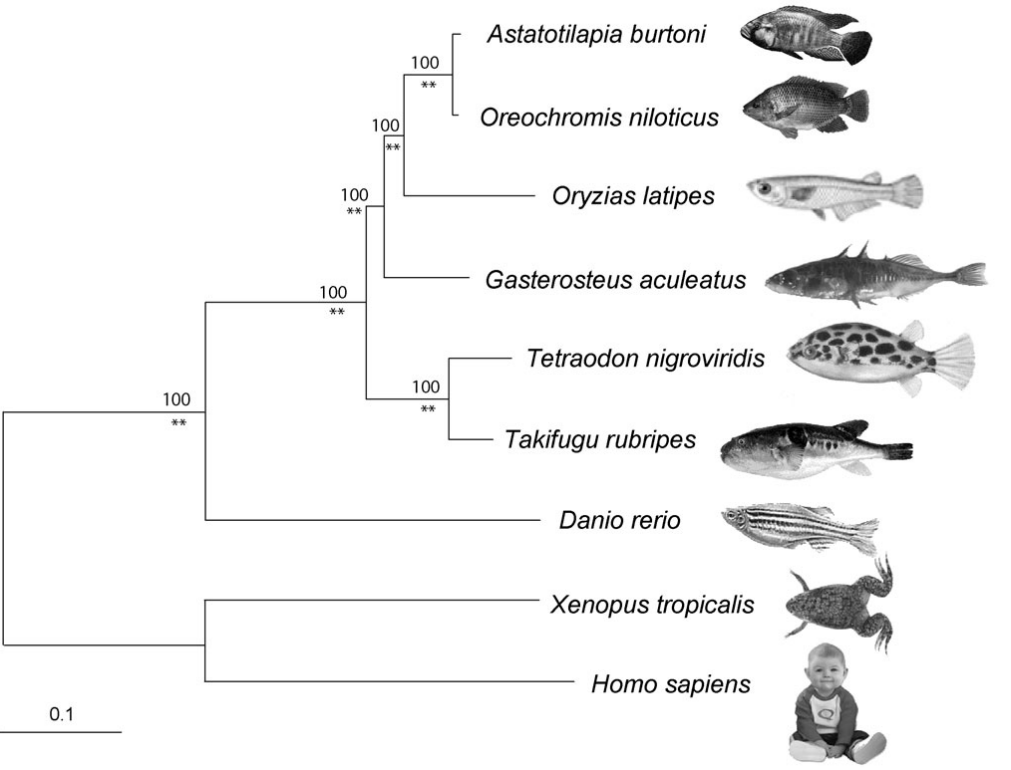
**Maximum likelihood tree based on 20,009 nucleotide positions of Hox genes**. Values above branches are Maximum Likelihood bootstraps; two asterisks indicate posterior probabilities of 1.00 as obtained by MrBayes 3.1.

Since there is no prior phylogenetic study including all of the model organisms that were part of our study, we performed a phylogenetic analysis in order to be able to trace gene loss events and the evolutionary history of the Hox clusters in an accurate phylogenetic framework. Based on alignments of coding regions, we selected 24 Hox genes (Table 1) for which orthologs had been identified both in human and *Xenopus tropicalis *and the complete dataset for seven teleost species: two cichlids (*Astatotilapia burtoni*, *Oreochromis niloticus*), medaka (*Oryzias latipes*), stickleback (*Gasterosteus aculeatus*), two pufferfishes (*Tetraodon nigroviridis*, *Takifugu rubripes*) and zebrafish (*Danio rerio*). When two teleost paralogs were available, we were careful to choose the more slowly evolving copy based on a preliminary tree to reduce the potentially detrimental effects of introducing noise into the dataset. We excluded positions that could not be aligned and concatenated 24 genes for a complete dataset of nine species and 20,009 basepairs. Modelgenerator [76] identified GTR + G (alpha = 0.53) as the best fitting model. With these parameters we ran PhyML [77] with 500 bootstrap replicates and MrBayes 3.1 for 1,000,000 generations, sampling every 10^th ^generation and with a burn-in of 5000. We obtained a fully resolved tree with maximal support for all nodes using both methods (Figure 2).

**Table 1:** Genes included in the phylogenetic analyses of teleost model species and the number of positions. We used only genes for which an ortholog in *Xenopus tropicalis *and *H. sapiens *was available, as well as the full sequences set for all teleost fishes for one paralog. Regions that could not be aligned were excluded from the analyses.

Cluster	Fish paralog	Positions included in analysis
HoxA	*evx1*	675
	*a13a*	791
	*a11a*	737
	*a9a*	639
	*a5a*	768
	*a4a*	624
	*a3a*	1106

HoxB	*b1b*	625

HoxC	*c13a*	921
	*c12a*	780
	*c11a*	915
	*c10a*	885
	*c9a*	783
	*c8a*	729
	*c6a*	630
	*c5a*	642
	*c4a*	762

HoxD	*evx2*	1236
	*d12a*	774
	*d11a*	700
	*d10a*	975
	*d9a*	633
	*d4a*	624
	*d3a*	1167

total	24 genes	20,009

*Danio rerio*, the only ostariophysian species for which a complete set of Hox clusters is currently available, is the sister group of the Neoteleosts, hence all other species included in this study. Within the Neoteleosts, two clades were recovered: firstly the pufferfishes, which form a monophyletic group and secondly, a clade consisting of stickleback, medaka and the cichlids, with a sister group relationship of *O. latipes *and the two cichlid species. The close relationship of cichlids with medaka has been previously described based on nuclear genes [78] and on ESTs [79].

### Gene loss and loss of microRNAs in the teleost Hox clusters

**Figure 3:**
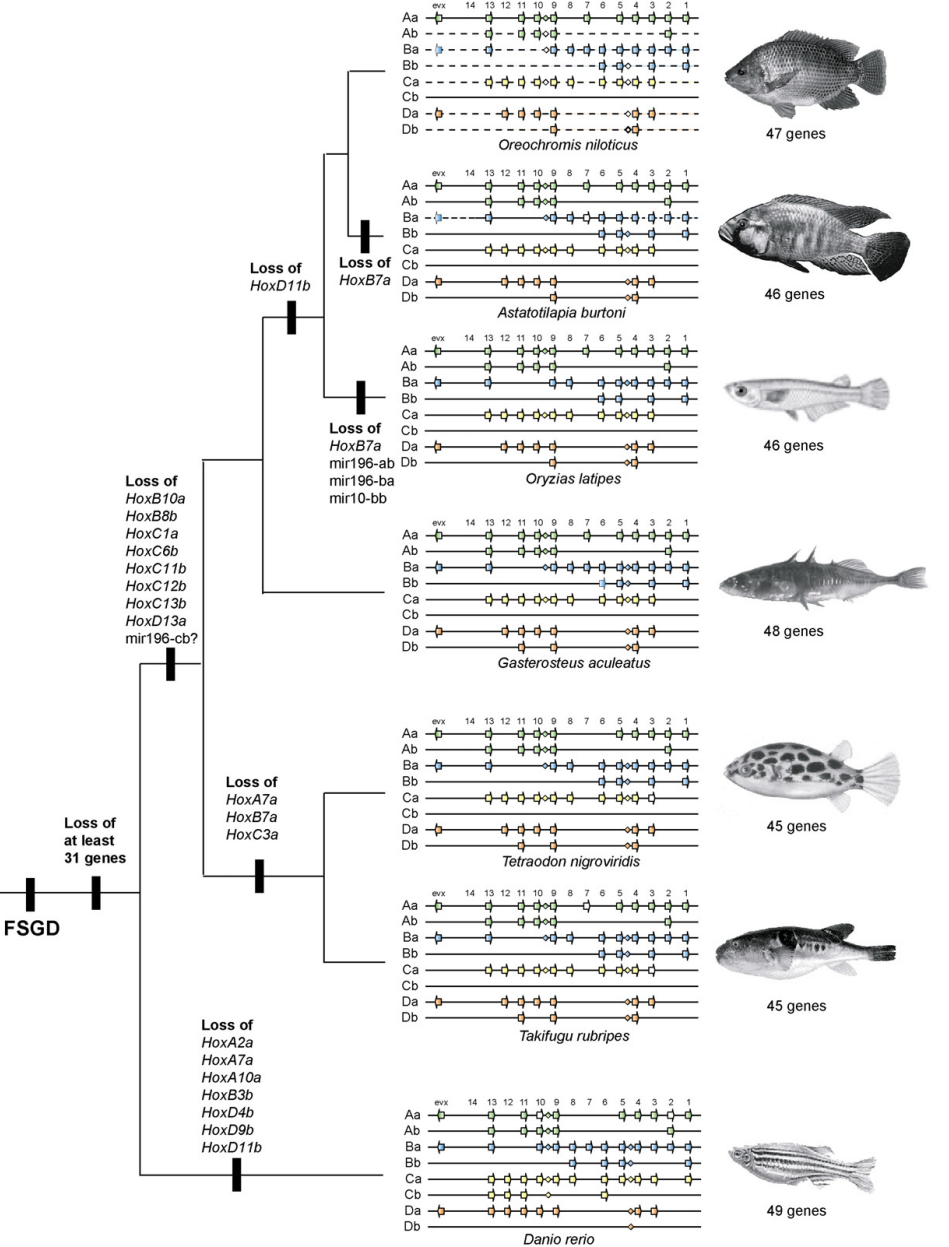
**Hox cluster of teleost model fish species and the event of gene loss plotted on a phylogeny**. Hox and Evx genes are shown as arrows, pseudogenes are shown without coloration and missing delineation indicates missing sequence data of most likely existing genes. MicroRNAs are drawn as small diamonds and were added according to our analyses. Data for *H. sapiens *were copied from [86] and the mir-10-db of *Danio rerio *according to [85].

We identified 46 functional coding sequences for Hox genes and one recent pseudogene in *Astatotilapia burtoni*. Based on the tree obtained, we traced events of gene loss and loss of microRNAs among these major fish model systems (Figure 3). The most salient gene losses that can be traced with confidence without complete data on basal teleosts and non-duplicated actinopterygians happened after the divergence of the Ostariophysii and Neoteleostei while most gene losses probably immediately followed the FSGD. Based on *Danio rerio*, the Ostariophysii have lost seven genes since the last hypothetical common ancestor with the Neoteleosts. During the evolution of the Neoteleosts eight Hox genes were lost. If we assume a divergence time of 290–304 mya between Ostariophysii and Neosteleostei [80,81] and an age of the genome duplication of 320–350 mya [19,20], at least thirty-one genes were lost within the 50 mya following the FSGD [31] and only 7–8 during the last 300 mya. This corroborates previous findings of high initial gene loss rates immediately following a large scale duplication event [82-84].

The pufferfish lineage lost three genes in the common lineage leading to *Takifugu *and *Tetraodon *(Figure 3). *Gasterosteus aculeatus *has the most complete set Hox genes, only the *hoxb6b *gene has not been identified so far, most likely due to a large sequence gap in the genomic contig, leaving open the possibility that it was not been lost. The loss of *hoxd11b *in both medaka and cichlids supports their monophyletic grouping in a parsimony framework. Both species also lost the *hoxb7a *gene, but due to independent events, as the existence of a functional *hoxb7a *gene in another cichlid species (*Oreochromis niloticus*) implies.

The sets of microRNAs in ostariophysian and neoteleost Hox clusters are similar but not identical. An equivalent to the mir196-Cb could not be identified in neoteleosts, which have lost the entire HoxCb cluster. So far, the assembly of the *D. rerio *genome is still incomplete and the HoxCb cluster is not contained in a single contig, therefore the identification of its neighboring genes and thus, the corresponding regions in Neoteleosts is not yet possible. *D. rerio *retains the mir10-Db copy between the *lunapark b *and *metaxin2 *genes, even though the hox genes in this genomic region have been lost [85]. In medaka, we were not able to identify mir196-Ab, mir196-Ba and mir10-Bb, even though sequences were complete and without gaps in these intergenic regions. Therefore these microRNAs might have been lost in the medaka lineage. In contrast to a previous study, we were able to identify mir196-Ab and mir196-Ba in the zebrafish clusters [86], probably due to increased sequence quality of the genomic sequence.

### Analyses of Conserved Non-coding Sequences (CNS)

We performed analyses of CNS using the program Tracker [52] with orthologous teleost Hox clusters. The datasets analyzed included 3 kb of additional sequence on both ends of the cluster. This rule was only changed for HoxCa clusters of both pufferfishes, where we used the complete genomic sequence up to the next downstream gene, *cbx5*, in order to be able include the pseudogene *hoxc3a *in this analysis. In the analysis of the HoxBb clusters we included upstream sequences to the end of the *TTLL6 *coding sequence to identify possible conserved sequences that surround *hoxb8b *in *Danio rerio *and may still exist in other fish, where this gene was lost. Also for HoxDb clusters in *Oryzias latipes *and *Astatotilapia burtoni*, the 5' region was extended until the beginning of the *lnb *gene, since we were not able to find a gene or pseudogene of *hoxd11b *with other methods in this species and we wanted to include any possible CNSs. For the pufferfishes, the 3' overlap had to be shortened because the intergenic region between *hoxd4b *and *mtx2 *is shorter than 3 kb. For a visual analysis, we also constructed mVista plots [87] based on LAGAN alignments [88] that are provided in Additional files 1, 2, 3, 4, 5, 6, 7. MicroRNAs are marked in green.

**Figure 4:**
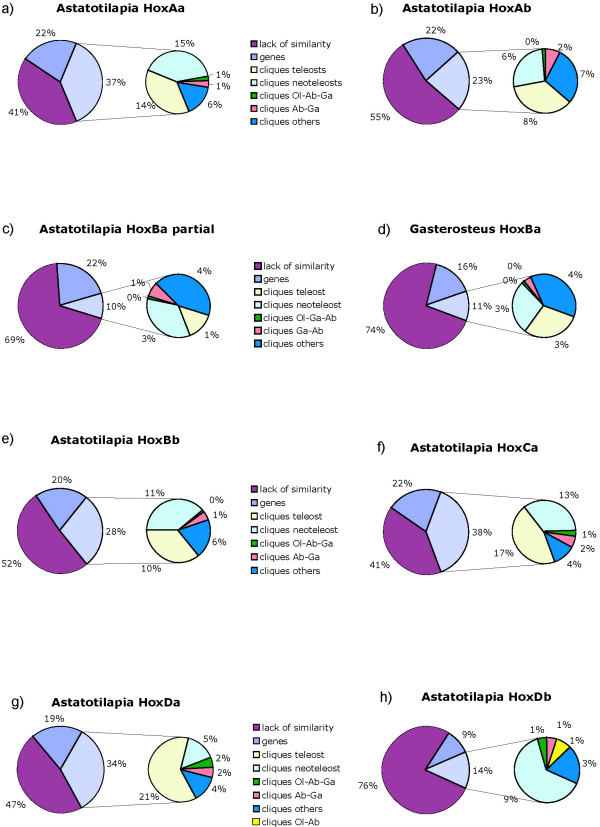
**Percentage of CNS within intergenic regions of the Hox clusters of Neoteleost fishes**. Starting from the complete length of analyzed sequence, we calculated the relative amounts of genes (including introns), PFC (as identified by Tracker) and marked the remaining sequence as "junk". The footprint cliques were further divided as shared by all six fish species included (teleost), shared by all species except zebrafish (neoteleost), shared by medaka, cichlid and stickleback (Ol-Ab-Ga) or shared by cichlid and stickleback (Ab-Ga). Against our expectations there were usually no or only very few cliques shared only between cichlid and medaka except for HoxDb.

**Figure 5:**
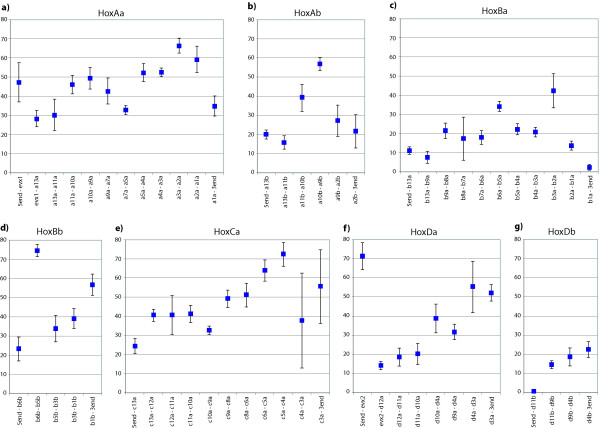
**"Proportional" analyses of the Hox clusters of Astatotilapia burtoni**. Large error bars for anterior regions of HoxCa cluster are explained by missing data from the pufferfish, which lost the hoxc3a gene.

For the HoxAa cluster, we obtained a total of 192 footprint cliques (FCs, clusters of conserved footprints forming a single alignment), which add up to a total length of 34.4 kb (37%) in *A. burtoni *(total cluster length 92.2 kb) (Figure 4). The sequence included all hox genes plus evx1. For the most part the identified CNS are teleost specific, i.e. present in all fish species included, (14%) or neo-teleost specific (15%, present in all species except zebrafish) (Figure 4). One of the teleost specific cliques contains mir196-Aa [see Additional File 1]. However, we found more CNS shared only between *A. burtoni *and *Gasterosteus aculeatus *than between *A. burtoni *and *Oryzias latipes*. This is in interesting disagreement with the phylogenetic hypothesis (Figure 2). Comparisons of the lengths of CNS relative to sequence length in intergenic regions along one cluster show a tendency to increase towards the anterior end of the cluster (Figure 5).

The HoxAb cluster is shorter (total length in *A. burtoni*: 33.2 kb) than the HoxAa cluster, contains only five genes and 53 PFCs could be identified, which cover a total of 7.6 kb (23%) of the *A. burtoni *HoxAb cluster. The average content of cliques in intergenic regions is lower than in the HoxAa cluster (Figure 4). While the proportion of coding sequence of the entire Hox cluster is about the same as in the HoxAa cluster, the CNS make up a much smaller portion of the total cluster length (37% in the HoxAa cluster, but only 23% in HoxAb cluster). Most of the CNSs are evolutionarily conserved and shared between all teleosts or at least between the neoteleosts. Also here we find more similarities between cichlid and stickleback than between cichlid and medaka. The footprint clique containing mir196-Ab did not identify a orthologous sequence for medaka, suggesting a lineage-specific loss of this microRNA in medaka [see Additional File 2].

For the HoxBa cluster, we included the partial cluster of *A. burtoni *(total length 135.0 kb) that we obtained in this study. For the statistical analyses we also compared data from stickleback (total length 233.3 kb) since it showed more similarities in terms of CNS to the cichlid than medaka. For all species except *A. burtoni*, the compared HoxBa cluster sequence included the sequence from *hoxb13a *to *hoxb1a*. In total, the analyses identified 311 footprint cliques, adding up to a total length of 13.3 kb in *A. burtoni *(10%) and 26.7 kb in *G. aculeatus *(11%). Comparing the relative proportions of coding sequences, CNS and non-conserved non-coding sequences are similar between *G. aculeatus *and *A. burtoni*, implying that the partial cluster is representative of the rest of this Hox gene cluster. Also, the distributions of amounts of CNS in the intergenic regions along the cluster are similar in both species (Figure 4). In both species, there is a long stretch of sequence between *hoxb13a *and *hoxb9a *that does not contain any footprint but gives BLAST hits to repetitive elements from the same species. The analyses revealed the existence of mir196-Ba in all teleosts – except for medaka – and mir10-Ba was found in all species but *A. burtoni*, due to missing sequence data [see Additional File 3]. While in terms of gene number the cluster is still complete, the CNS content is low (in comparison to other clusters), which is probably also an effect of the large intergenic regions.

The small HoxBb cluster had a total length of 29.2 kb, Tracker identified 70 FC, and the CNSs covered a total of 8.7 kb in *A. burtoni*. In contrast to its big "sister" HoxBa cluster, the CNSs make up a higher percentage of the cluster than the coding regions and the distribution of CNSs in the intergenic regions display a high density of conserved sites. The extremely short intergenic region between *hoxb6b *and *hoxb5b *results in the high peak seen in Figure 5d. Interestingly, the mir10-Bb could be identified in all species except again for *O. latipes *[see Additional File 4]. It is interesting to note that the medaka Hox clusters seems to lose microRNAs apparently more frequently than other fish lineages.

The HoxCa cluster contains 10 genes and has a complete length of 96.5 kb in *A. burtoni*. 38% of the clusters are CNS (37.0 kb) identified in the Tracker analysis, and make up more than the complete length of coding sequences (22%). The CNSs were mainly teleost and neoteleost-specific, and we could identify two microRNAs in all species: mir196-Ca and mir10-Ca [see Additional File 5]. In comparison with other clusters, the CNS density is high and there is also a slight increase in CNS length towards the anterior end of the cluster (Figure 5f).

For the HoxDa cluster, we also included the *evx-2 *gene and we could identify 113 footprint cliques. The sequence has a complete length of 53.9 kb in *A. burtoni *and the CNS take up 18.2 kb (34%) of it. Most of these are teleost specific (21%). The distribution shows a peak 5' of *evx-2*, where a highly conserved sequence has been described before [89]. Otherwise there is a noticeable trend for more CNS towards the anterior end of the HoxDa cluster [see Additional File 6].

The HoxDb cluster fragment analyzed for *A. burtoni *spanned 38.8 kb, reaching from the end of *lnpb *to 3 kb downstream of *hoxd4b*. We obtained 60 footprint cliques, the conserved sequences in *A. burtoni *have a total length of 5.3 kb (14%). The CNS are mainly neoteleost specific, due to the loss of the *Danio rerio *Hoxdb cluster, teleost specific CNS could not be detected. Our analysis also found the mir10-db in all species [see Additional File 7].

In order to be able to recognize general trends of sequence conservation of CNS in Hox clusters, we calculated the percentage of CNS for each intergenic region. Figure 5 shows the average of that percentage within the neoteleosts. We excluded zebrafish from this calculation, since the values were much lower due to evolutionary distance and the different gene setup of clusters made it difficult to include these data. Based on our analyses, the clusters with the highest percentage of CNS in their intergenic regions are HoxAa, HoxBb, HoxCa, and HoxDa (Figure 5a,d–f). It might have been expected therefore that HoxAa, HoxCa and HoxDa are also the clusters where most of the genes are conserved, while HoxBb only has retained four and yet, surprisingly, retained large sets of conserved CNS. Here, the high percentage of CNS (Figure 5d) is most likely caused by the high "gene density" of the cluster and the short intergenic regions. Whereas, the long intergenic regions of the HoxBa cluster "dilute" the CNS in this genomic region (Figure 5c). The anterior part of the HoxCa gene is very heterogenic in terms of CNS content due to gene loss in the pufferfishes (Figure 5e). HoxAb and HoxDb have also lost genes (by comparison to their paralogous clusters) and, correspondingly, CNS (Figure 5b,g). A general interesting trend that is observed in all clusters (except HoxAb) is a maximal peak of CNS towards the anterior end of the clusters (Figure 5).

## Discussion

We screened the BAC library of *A. burtoni *for Hox-positive clones and identified clones with complete clusters by PCR of the 5' and 3' most genes. Since obtained sequence data included surrounding genes for most clusters, we can be certain that our analysis misses only maximally four genes of the HoxBa cluster. Through PCR, sequence data for those genes (*hoxb4a*, *hoxb3a*, *hoxb2a and hoxb1a*) and partial non-coding data of this region were also obtained and, therefore, we can safely assume that these genes are clustered also in *A. burtoni*. Our analyses of the non-coding area of the partial HoxBa cluster of *A. burtoni *in comparison with the complete cluster of *G. aculeatus *shows that the features of this Hox cluster of these two species are similar. The Hox gene content is almost identical to that of *Oreochromis niloticus *[40]. There are two exceptions: we were not able to identify any trace of a *hoxd11b *gene in the BAC library of *A. burtoni*. A previously described sequence, which claimed to be from a cichlid [40] is almost identical to *Tetraodon nigroviridis *(169 of 171 base pairs identical), including a part of the additional intron this gene has acquired. This suggests that this is not an *Oreochromis *sequence (AY757355) as claimed by Santini and Bernardi [40] but rather is indeed simply previously published *Tetraodon nigroviridis *sequence instead. We strongly suspect that a large portion of the *Oreochromis *sequences of that study [40] were taken from an unpublished data set of the Meyer laboratory which was not collected by S. Santini but published fraudulently under her name. The proposed cichlid genome projects on *Oreochromis *and *Astatotilapia *will also aid in the clarification of this matter.

The other difference between the cichlids is the existence of a stop codon in the sequence of *hoxb7a *of *A. burtoni *while the coding sequence of *Oreochromis niloticus *is still intact [[40], Hoegg et al. unpublished data]. This implies not only that *hoxb7a *was lost independently in different lineages of fish such as in the lineages leading to pufferfish or medaka, but also in at least part of the cichlid fish radiation, suggesting that it is not essential and can be lost easily and repeatedly. However, some selective forces apparently did prevent it from being deleted for probably hundreds of millions of years since the fish-specific genome duplication [20]. More detailed analyses of its expression, the exact phylogenetic timing of gene loss and its possible implications for speciation in haplochromine cichlids will be needed to investigate this further. Differences in gene content of the Hox gene clusters, the essential developmental toolkit, that differentiate two species of closely related African cichlid fish species is a rather unexpected finding.

We performed a phylogenetic analysis based on a dataset of 24 Hox genes from seven fish species and two outgroup species (human and frog) and obtained a single, highly supported tree (Figure 2), which shows a monophyletic group of *G. aculeatus*, *O. latipes *and the cichlids *A. burtoni *and *O. niloticus*. The close relationship of Beloniformes (*O. latipes*) and Perciformes (*A. burtoni/O. niloticus*) is in agreement with recent molecular phylogenies [78,79] and rejects the monophyly of Smegmamorpha, a clade that contains Beloniformes and Gasterosteiformes but not Perciformes. Even though the Order Perciformes is not monophyletic itself [62], more data including more species will be required to resolve the complete phylogeny. This will be necessary in the future to make assumptions about genomic evolution in the neoteleost fishes within the correct phylogenetic framework. A new phylogeny and a new classification of the highly diverse clade Percomorpha is especially required. For our species set used, we are confident in the tree, especially since it is also fully congruent with the inferred gene loss patterns (Figure 2). Only the position of *G. aculeatus *cannot be determined with certainty by the parsimony approach of gene losses.

Also the Hox phylogeny indicates a close relationship of cichlids and medaka to the exclusion of other orders in the Percomorpha such as the pufferfishes and the sticklebacks. Interestingly, the analyses of CNS, however, show consistently a higher similarity between *G. aculeatus *and *A. burtoni *than between *O. latipes *and *A. burtoni *(Figure 4). CNS shared only between *O. latipes *and *A. burtoni *where consistently too few to show, except for *HoxDb*. Also, of nine miRNAs contained in the other neoteleostean species, three were lost in the *O. latipes *cluster (Figure 3). In general, we find a high variability of gene content within teleost fish, no two of the species examined so far had the same gene content in their clusters. This might be due to the redundancy that was created by the FSGD, and that still permits gene loss without major consequences on the bodyplan. In tetrapods, it is assumed that the Hox gene setup is more conserved. However, data from the frog *Xenopus tropicalis *[31] and from the coelacanth *Latimeria menadoensis *[90] show that there is variation also among sarcopterigians. When more tetrapod lineages are examined, it will become more clear if and how much more variable fish clusters are.

The finding of greater similarity between sticklebacks and cichlids in regulatory elements suggests however, that the medaka genome evolves at a higher rate, at least in non-coding sequences. Why this should be so deserves further attention. The coding sequences also show a slightly accelerated rate of evolution in comparison to other neoteleost species (Figure 2). The increase in evolutionary rate rather seems to be a Hox-specific trend; a study on differential rates of duplicate genes more often identified slower evolutionary rates in medaka rather than accelerated ones [44]. Since the sequences of the *O. latipes *Hox clusters are not directly taken from a genome sequencing project but from sequenced BAC clones with only few, small gaps, possible artifacts due to potential assembly problems of the medaka genome can be excluded.

The analyses of CNS of *A. burtoni *showed as well that the major part, or at least the longest part, of the potential regulatory elements is conserved between all teleost species included in this study, or at least between neoteleost species. This can also be seen as an indication that the analysis parameters have been chosen correctly, are conservative and do not tend to overestimate the number of phylogenetic footprints. This also indicates that even though the gene content in Hox clusters in fish is more variable than previously thought, the main regulatory elements are highly conserved. That gene loss and gain even in such important genes such as Hox genes, even among relatively closely related species, might imply that the putative paramount importance of regulatory evolution in bringing about phenotypic change during evolution is not as great as generally believed.

We could also show that there is a trend towards more CNS in the anterior portion of the cluster (Figure 5) which has been described before for the HoxAa cluster based on a different analytical method [59]. Knockout studies on other vertebrates showed that a complete knockdown for paralogy group 1 in *Xenopus *results in serious developmental defects [91], and mutations in HoxA1 in humans are linked with Bosley-Salih-Alorainy syndrome effecting delayed development, eye movement and formation of the cranial nerve VIII [92]. Several mutations for posterior genes have been described in HoxA13 (Hand-foot-genital syndrome [93]) and HoxD13 (Synpolydactyly [94]). This might imply that anterior Hox genes are usually buffered by functional redundancy of paralogs and any mutation in the CNS in those anterior Hox-clusters would be expected to be severe and most likely lethal. Therefore, the regulation of anterior Hox genes would be expected to be more important for the patterning and survival of an embryo than those of posterior genes, which do not affect the brain and head development to the same extent. Similar conclusions were drawn from the observation that among the vertebrates, sequence divergence between posterior genes is higher than between more anterior genes, an effect termed "laxitas terminalis" [95]. The authors of this study suggest that posterior genes are not linked to basal vertebrate functions but rather fulfill lineage specific functions. In a comparison among all posterior Hox genes among more distantly related phyla, it was found that deuterostome posterior genes are evolving faster than their protostome counterparts as well as the deuterostome anterior genes [96]. This "posterior flexibility" also indicates a higher conservation of the anterior part of the clusters.

Our study also shows that different Hox clusters are evolving with distinct patterns in different evolutionary lineages, even though an overall evolutionary trend can be observed: that after duplication, one cluster retains more genes and also the regulatory elements that go with them while the paralogous cluster loses genes and conserved elements concomitantly more rapidly and possibly more easily due to relaxed constraints. Our data also demonstrate clearly that the loss of Hox genes in teleost clusters is an ongoing process that occurred even within the last seven million years within the cichlid family (*hoxb7a*). A close comparison among different closely related genes might also provide insight into species-specific differences and the potential influence of regulatory evolution on different cichlid species.

## Conclusion

Hox clusters in fish are more variable in gene content than expected and also, each cluster has its own characteristics in terms of absolute length and content of CNS. While genes have continuously been lost, somewhat surprisingly most microRNAs remained unchanged (with the notable exception in the medaka lineage). The CNSs form a large portion of Hox clusters, usually even more basepairs than the coding regions and are, typically, conserved over very long evolutionary time spans. Their distribution is not constant along the cluster but the maximum frequency of occurrence is usually towards the anterior end, implying stronger selection on the anterior Hox gene expression patterns, while the more posterior Hox genes are more free to vary.

## Methods

### DNA extraction and PCR

DNA was extracted from muscle tissue or fins from specimens stored at -80°C following a standard phenol-chloroform protocol. PCRs were performed in 25 μl reactions using 0.5–1 units of RedTaq (Genaxxon, Germany) and the corresponding reaction buffer (10 mM Tris-HCI (pH 9.0 at 25°C), 50 mM KCl, 1.5 mM MgCl_2_, 0.1% Triton X-100), 1 mM additional MgCl_2_, 0.6 mM dNTPs (Genaxxon, Germany), 0.4 μM primers and for long fragments 0.1 unit of Pwo polymerase (Fermentas, Germany) was added. PCR used an initial denaturation step at 94°C for 3 minutes, followed by 35 cycles with 20 sec at 94°C, 40 seconds at 58°C and 2.5 minutes at 68°C, and a final extension step at 68°C for 7 minutes. PCR products were checked on 1% agarose gels running in 1× TAE buffer containing 0.05% ethidium bromide. PCR fragments were purified directly via spin columns (PEQLAB, Germany) or were cut from preparative agarose gels (1%) using the gel extraction kit (QIAGEN, Germany).

### Primer design

Primers were designed in conserved regions of the coding sequence, preferably the forward primer at the beginning of the first exon and the reverse primer in the second exon outside the homeodomain, so the PCR fragment would cover the intron and yield specific fragments for screening of the BAC library. (Primer sequences are provided in Additional file 8).

### Screening of the *Astatotilapia burtoni *BAC library and plasmid preps

The BAC library was spotted on four filters containing 18,432 clones each. The screening using chemiluminescence was conducted according to Lang et al. (2006). Positive clones were picked from the library and grown overnight in Luria Broth Base medium (Invitrogen™) containing 12.5 μg/ml chloramphenicol. Plasmids were isolated using a modification of a standard plasmid miniprep protocol [97]. Gene content of the BAC clones were confirmed by PCR for 5' and 3' most Hox/Evx genes. For size estimation, BACs were digested with NotI and ran on a pulse-field gel apparatus.

### Shotgun sequencing of BAC clones

BAC clone DNA was isolated from each preparation, and then sheared into random fragments of approximately 3 kb by repeated passage through a narrow aperture using a Hydroshear device. These fragments were repaired to blunt ends using T4 polymerase and Klenow fragment, and then a narrow distribution of sizes was selected from an agarose gel. These fragments were ligated into plasmid vector, introduced into *E. coli *by electroporation and then plated on nutrient agar. A random selection of these clones was processed for sequencing reads from each end using rolling circle amplification of the plasmids, sequencing reactions using BigDye terminators (ABI), cleanup using solid phase reversible immobilization (SPRI), then sequence determination on an ABI 3730 × l automated DNA sequencer.

### Sequence assembly

Raw sequences were trimmed for vector sequences and sequence quality was scored with Phred. Contigs were assembled automatically using Sequencer™ using a minimal overlap of 17 nucleotides and a minimal identity of 85% and refined and corrected manually. Sequence gaps were closed by PCR using sequence specific primers designed with Primer3.

### Annotation

Genes were annotated manually by pairwise BLAST and based on alignments of available sequences from other species. We also performed BLAST searches against the EST sequences available for *A. burtoni *as well as two other haplochromine cichlids (*Haplochromis chilotes*, *Haplochromis *sp. 'red tail sheller') [98], especially for a better annotation of adjacent non-Hox genes. BAC clone sequences were submitted to GenBank (accession numbers EF594310–EF594316).

### Database searches and phylogenetic analyses

Complete Hox cluster sequences were downloaded from GenBank (*Homo sapiens, Oryzias latipes, Takifugu rubripes*) [99], the Joint Genome Institute (*Xenopus tropicalis*) [100], Ensembl (*Danio rerio *(Zv6), *Gasterosteus aculeatus *(BROAD S1)) [101], and Genoscope (*Tetrodon nigroviridis*) [102] [for accession numbers see Additional file 9]. Coding sequences were aligned based on their amino acid sequences with their respective orthologs using ClustalW as implemented in Bioedit. Regions that could not be aligned with confidence were omitted from the phylogenetic analyses [the final alignment is given in Additional file 10]. For the concatenated datasets, only genes that were available for both tetrapod outgroup species were used (*H. sapiens and X. tropicalis*) as well as for all seven fish species (*A. burtoni*, *O. niloticus*, *O. latipes*, *G. aculeatus*, *T. rubripes*, *T. nigroviridis*, *D. rerio*). For genes with two paralogs in fish we selected the slower evolving copy to avoid additional noise in the dataset. We performed a Maximum Likelihood analysis using PhyML [77]with 500 bootstrap replicates as well as an analysis based on Bayesian Inference with the MrBayes 3.1 [103] software for 1,000,000 generations and a burn-in of 5,000 with sampling every 10^th ^generation.

### Analyses of non-coding sequences

Genomic regions were prepared for analyses including 3 kb of sequence upstream of the first Hox gene and downstream of the 3'-most Hox gene. For HoxBb clusters, sequences between *ndp52 *(5') and *scap1 *(3') were used since *D. rerio *also has a Hoxb8b gene, which was lost in all neoteleosts for which this genomic information is available, since we wanted to avoid losing sequence information. In both pufferfish HoxCa clusters, sequences up to the next downstream gene (*cbx5*) were used because this lineage has lost hoxc3a. For species that had lost the *hoxd11b *gene (*Astatotilapia burtoni, Oryzias latipes*) sequence data until *lnb *(*lunapark b*) were included. For an overview as well as for a visual display, we used mVISTA [87] based on LAGAN multiple alignments [88]. For more detailed analyses, we used the program Tracker [52], using more stringent than default parameters (minimal BlastZ score 2000, minimum identity 85%) since the sequences analyzed here are more closely related than those used in previous studies. Phylogenetic footprint cliques obtained through Tracker were checked carefully for double hits of the same alignments and microsatellites that were eliminated. Footprint cliques containing microRNAs were identified.

## Authors' contributions

This study was conceived by SH, JLB and AM. The laboratory work was done by SH, except for shotgun sequencing of the BAC clones and sequence assembly, which was conducted by JVK. In silico analyses were performed by SH. The manuscript was drafted by SH and read and revised by all other authors.

## Supplementary Material

Additional file 1 Vista plot of HoxAa cluster based on LAGAN alignment with reference sequence *Astatotilapia burtoni*. Click here for file

Additional file 2 Vista plot of HoxAb cluster based on LAGAN alignment with reference sequence *Astatotilapia burtoni*. Click here for file

Additional file 3 Vista plot of HoxBa cluster based on LAGAN alignment with reference sequence *Gasterosteus aculeatus*. Click here for file

Additional file 4 Vista plot of HoxBb cluster based on LAGAN alignment with reference sequence *Astatotilapia burtoni*. Click here for file

Additional file 5 Vista plot of HoxCa cluster based on LAGAN alignment with reference sequence *Astatotilapia burtoni*. Click here for file

Additional file 6 Vista plot of HoxDa cluster based on LAGAN alignment with reference sequence *Astatotilapia burtoni*. Click here for file

Additional file 7 Vista plot of HoxDb cluster based on LAGAN alignment with reference sequence *Tetraodon nigroviridis*. Click here for file

Additional file 8 Primers used in this study Click here for file

Additional file 9 Accession numbers of sequences used in this study Click here for file

Additional file 10 Phylogenetic alignment used in this study Click here for file
