# Supplementary figures and images for: Comparative phylogenomic analyses of teleost fish Hox gene clusters: lessons from the cichlid fish Astatotilapia burtoni
Source: BMC Genomics. 2007 Sep 10;8:317. doi: 10.1186/1471-2164-8-317 (PMC2080641; doi:10.1186/1471-2164-8-317)

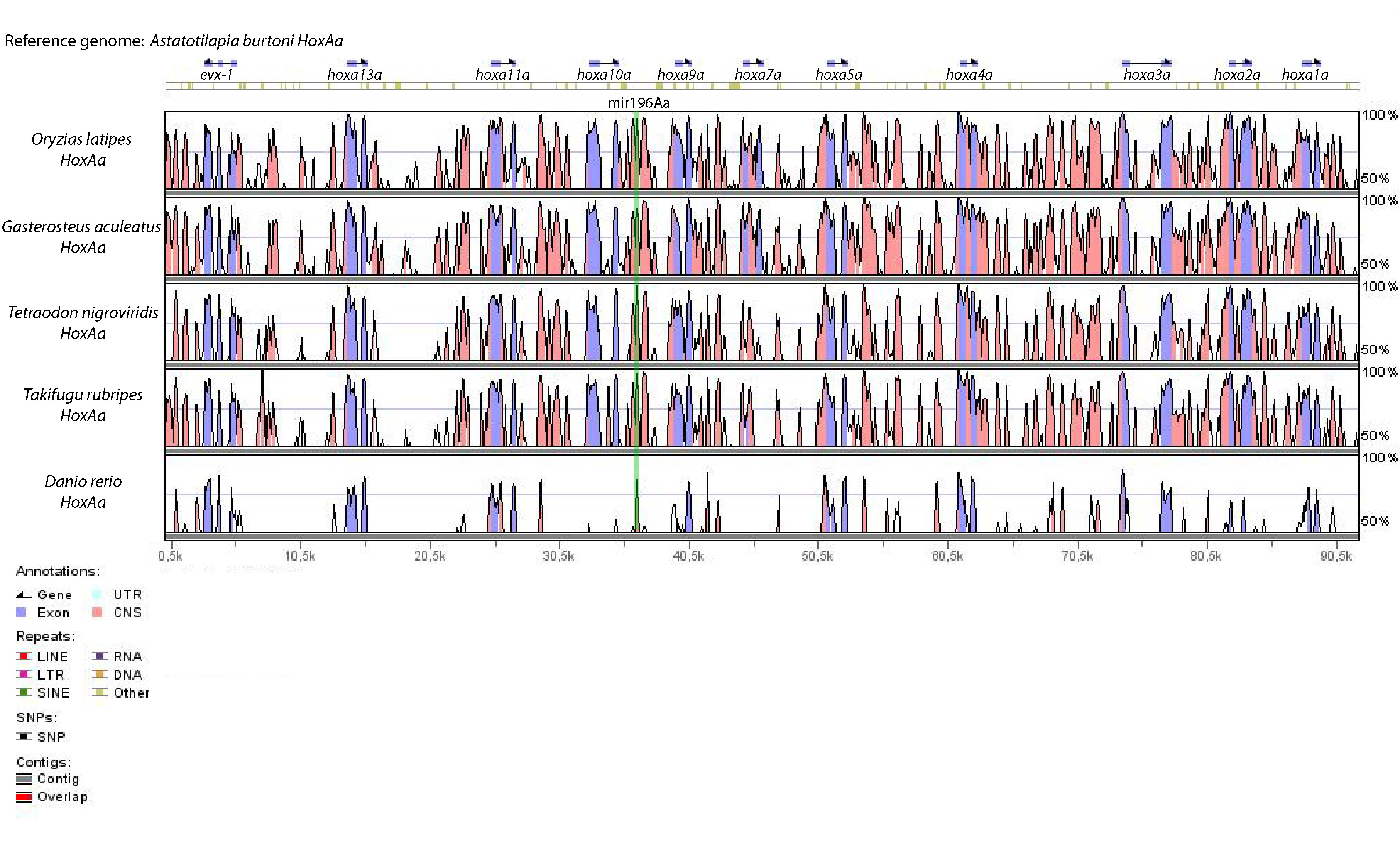

Supplement: Additional file 1 — Vista plot of HoxAa cluster based on LAGAN alignment with reference sequence Astatotilapia burtoni. [file 1471-2164-8-317-S1.jpeg]

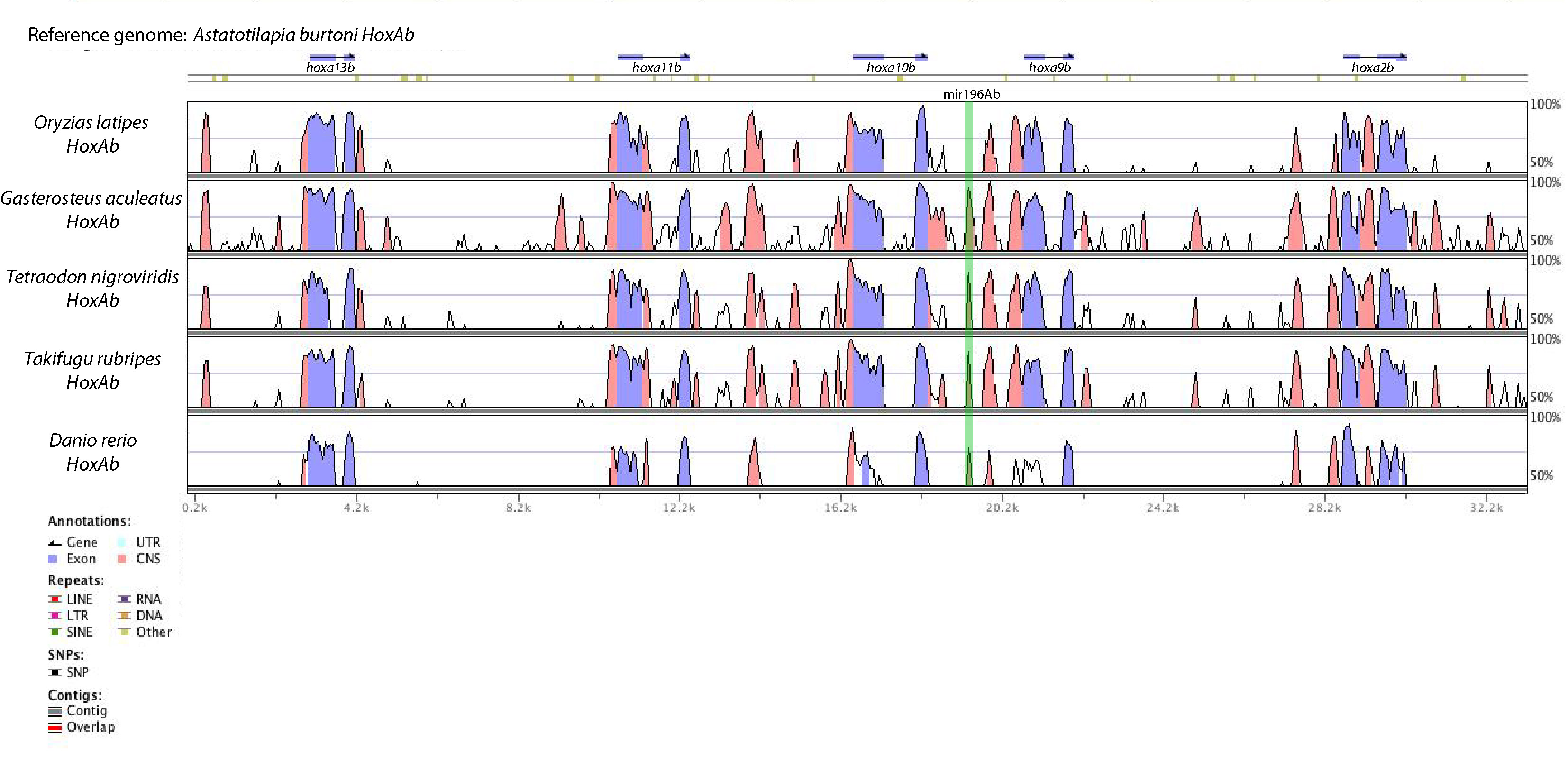

Supplement: Additional file 2 — Vista plot of HoxAb cluster based on LAGAN alignment with reference sequence Astatotilapia burtoni. [file 1471-2164-8-317-S2.jpeg]

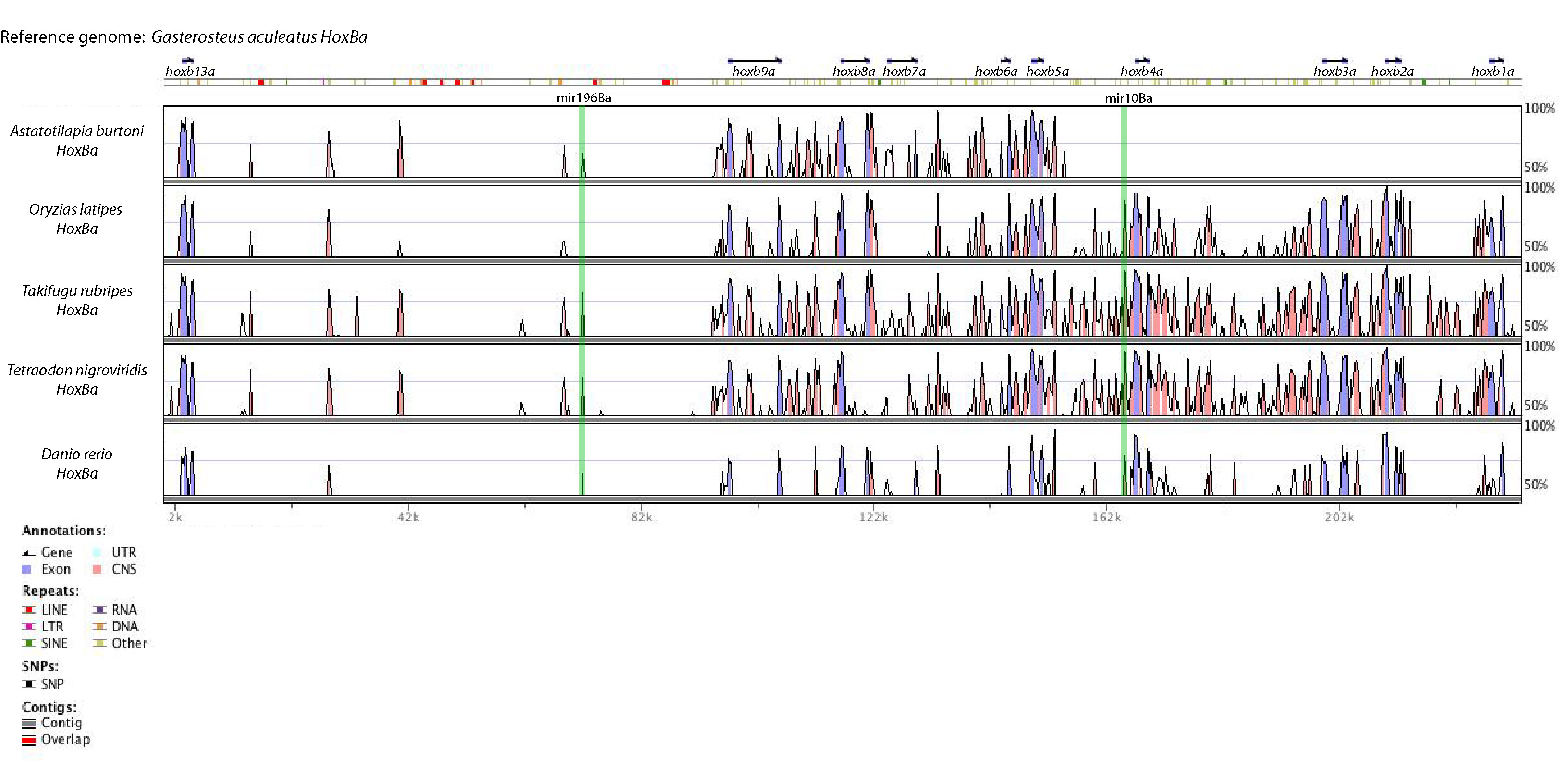

Supplement: Additional file 3 — Vista plot of HoxBa cluster based on LAGAN alignment with reference sequence Gasterosteus aculeatus. [file 1471-2164-8-317-S3.jpeg]

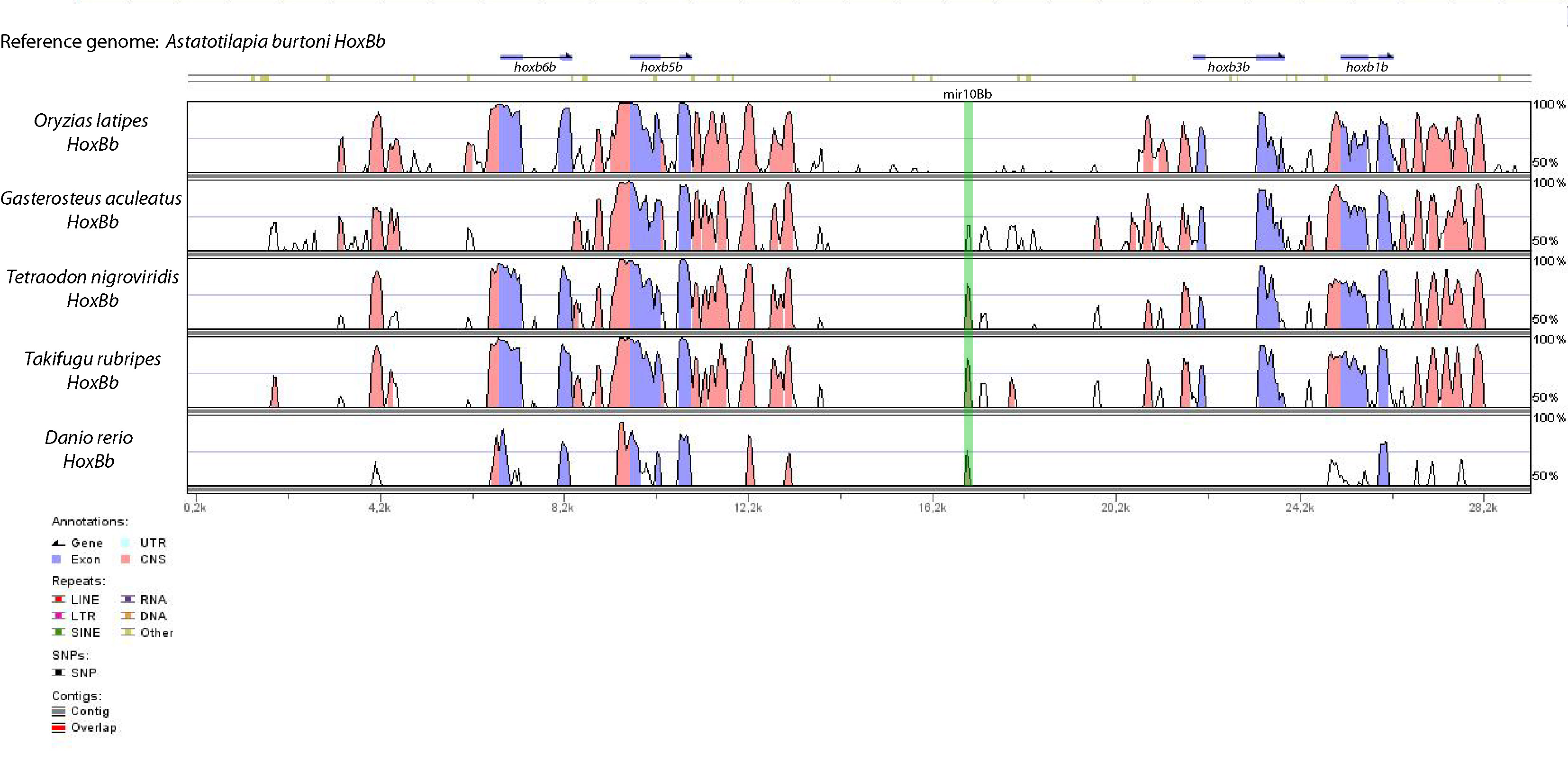

Supplement: Additional file 4 — Vista plot of HoxBb cluster based on LAGAN alignment with reference sequence Astatotilapia burtoni. [file 1471-2164-8-317-S4.jpeg]

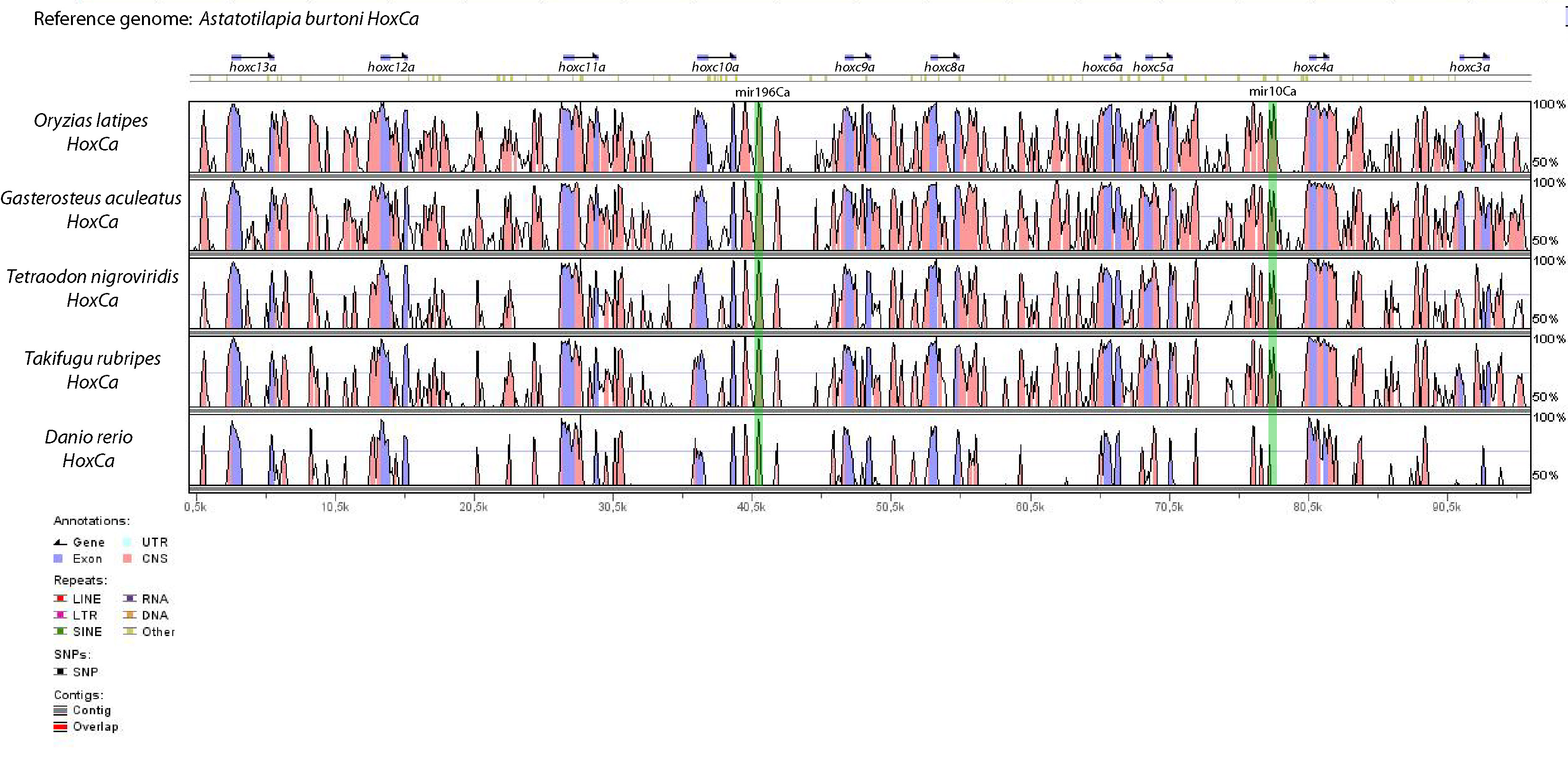

Supplement: Additional file 5 — Vista plot of HoxCa cluster based on LAGAN alignment with reference sequence Astatotilapia burtoni. [file 1471-2164-8-317-S5.jpeg]

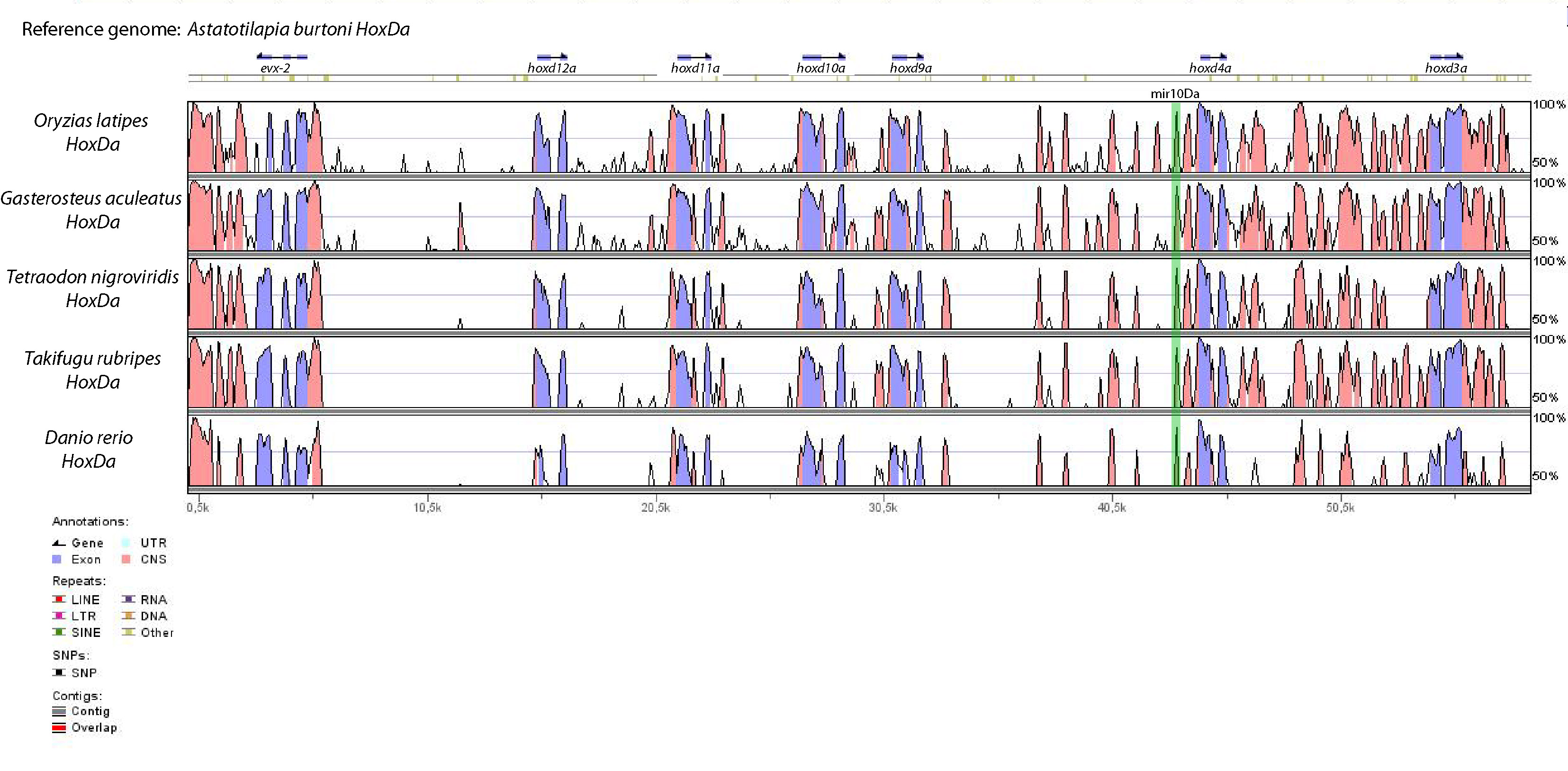

Supplement: Additional file 6 — Vista plot of HoxDa cluster based on LAGAN alignment with reference sequence Astatotilapia burtoni. [file 1471-2164-8-317-S6.jpeg]

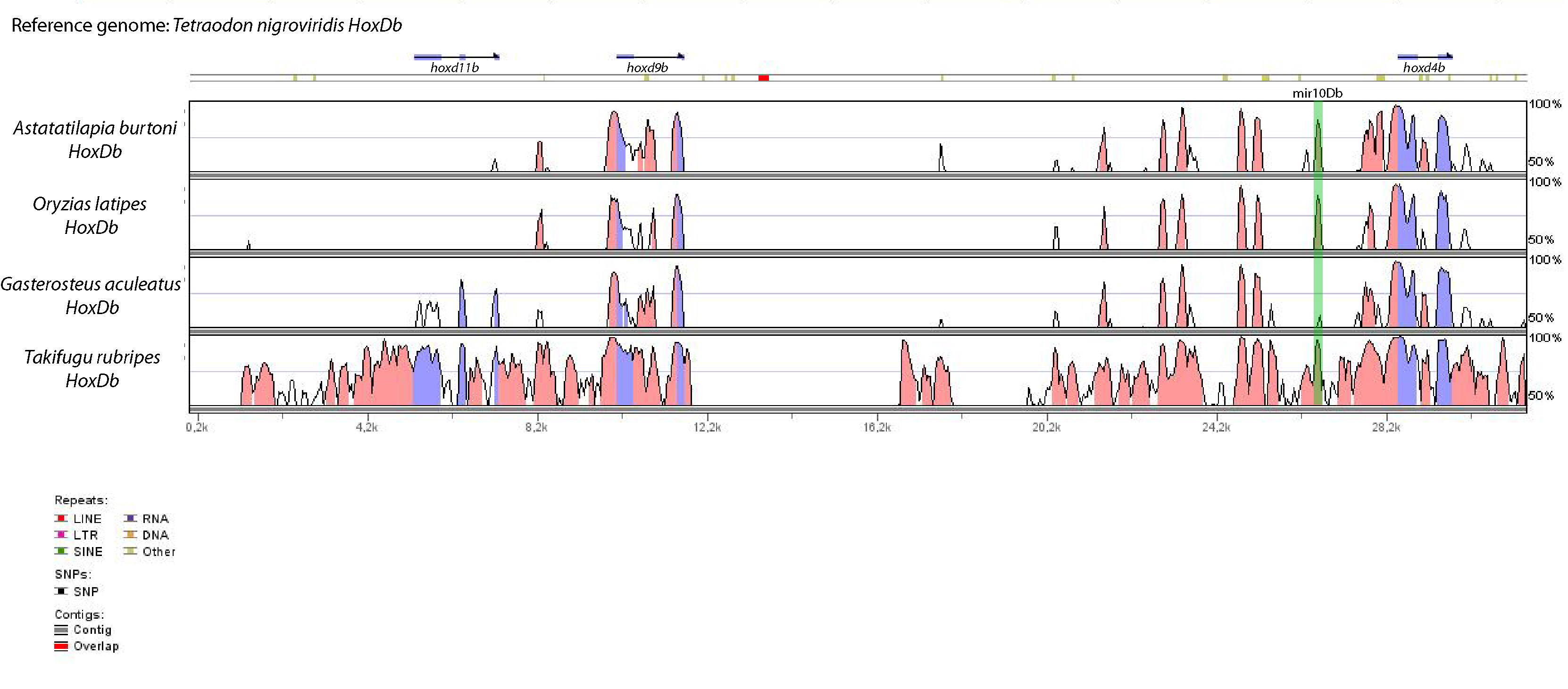

Supplement: Additional file 7 — Vista plot of HoxDb cluster based on LAGAN alignment with reference sequence Tetraodon nigroviridis. [file 1471-2164-8-317-S7.jpeg]
