# Supplementary material for: Comparative phylogenomic analyses of teleost fish Hox gene clusters: lessons from the cichlid fish Astatotilapia burtoni
Source: BMC Genomics. 2007 Sep 10;8:317. doi: 10.1186/1471-2164-8-317 (PMC2080641; doi:10.1186/1471-2164-8-317)
Supplement: Additional file 8 — Primers used in this study [file 1471-2164-8-317-S8.doc]

Table S1: Primers used in this study:

| Name | Cluster | Gene | Sequence |
| --- | --- | --- | --- |
| Hoxa13a.Ex1.For2 | Aa | a13a | AGT GAG CAA GAA CAT GGA GG |
| Hoxa13a.Ex2.Rev2 | Aa | a13a | ACC ATA TCG TGA CCT GTC GC |
| Hoxa1a.Ex1.For2 | Aa | a1a | TGC AGC TCT AAC TAT GGA CC |
| Hoxa1a.Ex2.Rev2 | Aa | a1a | CTG ACT GCT GTC CGA GGA TG |
| Hoxa13b.Ex1.For | Ab | a13b | CAA GGA GCA ACC ACA GTC AA |
| Hoxa13b.Ex2.Rev | Ab | a13b | GTC ACT TGT CTC TCT GTC AGG |
| Hoxa9b.For.Ex1 | Ab | a9b | CCT GTG TTT GAC GGG ATT ACC |
| Hoxa9b.Rev.Ex2 | Ab | a9b | GTT GCA GGA TTT GAT GCT TTG T |
| Hoxa2b.Ex1.For | Ab | a2b | CTC GCT GAG TGC CTG ACA TC |
| Hoxa2b.Ex2.Rev | Ab | a2b | TTT CCA CTC GCC TCG GTC TG |
| Hoxb13a.Ex1.For2 | Ba | b13a | CGT GCC GCA YCA RAG CTC |
| Hoxb13a.Ex2.Rev | Ba | b13a | TGC TCT TCA CTT TGG CCA CG |
| Hoxb5a.Ex1.For2 | Ba | b5a | ATG AGC TCT TAC TTT GTA ACT CG |
| Hoxb5a.Ex2.Rev | Ba | b5a | TTG ATC TGG CGC TCC GTG AG |
| Hoxb3a.Ex2.For2 | Ba | b3a | AGT GTC TTA CTC CAT GTC CAC TGC CTA |
| Hoxb3a.Ex2.Rev1 | Ba | b3a | CTA CAG GTG AGT GAG CTT GG |
| Hoxb2a.Ex1.For | Ba | b2a | CCC GTC GTC AAC TGC CTC TG |
| Hoxb2a.Ex2.Rev | Ba | b2a | GCT CAA AGC CTC CCG TGT CG |
| Hoxb1a.Ex1.For4 | Ba | b1a | CCG GGG CCT ACT GCG GG |
| Hoxb1a.Ex2.Rev2 | Ba | b1a | GAG TGG TCA GTG TCC TCC A |
| Hoxb6b.Ex1.For | Bb | b6b | CGT GGA TGC AGA GGA TGA AC |
| Hoxb6b.Ex2.Rev | Bb | b6b | CCT AAA TAA ACG CAC GAG CAC |
| Hoxb1b.Ex1.For | Bb | b1b | ACT GGA TGA AAG TSA AGA GGA |
| Hoxb1b.Ex2.Rev | Bb | b1b | AAC CAG ATK TTW ACC TGC GT |
| Hoxc13a.Ex1.For | Ca | c13a | CAC CAG GGC TCC GTC TAC TC |
| Hoxc13a.Ex2.Rev | Ca | c13a | CTT GCT GCA TAC TCT TTC TCC |
| Hox.c9a.Ex1.For | Ca | c9a | GAC CTA CAC GGA TTA TCT GTA |
| Hox.c9a.Ex2.Rev | Ca | c9a | TCT TGT AAG GTA CAT ATT GAA C |
| Hoxc3a.Ex1.For | Ca | c3a | CAT GAC CTC CTC TTC TCG TGT |
| Hoxc3a.Ex2.Rev | Ca | c3a | GGT GAT GAC TAT CTA TGC TTG |
| Evx2.Ex2.For | Da | evx2 | GAG AAC AAA TCG CAA GAC TGG |
| Evx2.Ex3.Rev | Da | evx2 | ATG GAA GTG GCG AAA GGT GAC |
| Hoxd9a.Ex1.For | Da | d9a | AAT GTC TTC CAG TGG CAC TC |
| Hoxd9a.Ex2.Rev | Da | d9a | CAG ATC TTC ACT TGT CTC TC |
| Hoxd3a.Ex1.For2 | Da | d3a | TAC ACC TAC CCC AAA CCA GAC |
| Hoxd3a.Ex2.Rev2 | Da | d3a | GAT CTG AGC GGC ACA GCT G |
| Hoxd4b.Ex1.For2 | Db | d4b | CGC TCA GGT GCA GAG GAA |
| Hoxd4b.Ex2.Rev2 | Db | d4b | CAC ACC TTC ACC TGC CGC TC |
