# Supplementary material for: Comparative phylogenomic analyses of teleost fish Hox gene clusters: lessons from the cichlid fish Astatotilapia burtoni
Source: BMC Genomics. 2007 Sep 10;8:317. doi: 10.1186/1471-2164-8-317 (PMC2080641; doi:10.1186/1471-2164-8-317)
Supplement: Additional file 9 — Accession numbers of sequences used in this study [file 1471-2164-8-317-S9.doc]

Table S1: Accession numbers of sequences used in this study

|  | *Danio rerio* | *Astatotilapia burtoni* | *Oryzias latipes* | *Gasterosteus aculeatus* | *Takifugu rubripes* | *Tetraodon nigroviridis* |
| --- | --- | --- | --- | --- | --- | --- |
| HoxAa | AC107365 | EF594313 | AB232918 | groupX: 9851119-9940576 | DQ481663 | chr21: 1370001-1470000 |
| HoxAb | AC107364 | EF594311 | AB232919 | groupXX: 9700000-9745000 | DQ481664 | chr8: 6600000-6633000 |
| HoxBa | BX927395, AL645782 | EF594310 | AB232920 | groupXI: 1514000-1750000 | DQ481665 | Un_random: 38022000-38183000 |
| HoxBb | AL645798 | EF594314 | AB232921 | groupV: 4590000-4630000 | DQ481666 | chr2: 1418500-1440500 |
| HoxCa | BX465864, BX005254 | EF594312 | AB232922 | groupXII: 11535000-11680000 | DQ481667 | chr 9: 4178000-4304000 |
| HoxDa | BX322661 | EF594315 | AB232923 | groupXVI: 9785000-9855000 | DQ481668 | chr2: 11072000-11123000 |
| HoxDb | -- | EF594316 | AB232924 | groupVI: 16153000-16185200 | DQ481669 | chr17: 9565000-9596500 |
